# Orbital leiomyoma originating from Muller muscle: A case report and review of the literature

**DOI:** 10.1016/j.ijscr.2024.110466

**Published:** 2024-10-16

**Authors:** Rakan Alsaad, Hind M. Alkatan, Hatim Khoja, Adel Alsuhaibani

**Affiliations:** a Department of Ophthalmology, College of Medicine, King Saud University, Riyadh, Saudi Arabia; b King Saud University Medical City, King Saud University, Riyadh, Saudi Arabia; c Department of Pathology and Laboratory Medicine, College of Medicine, King Saud University, Riyadh, Saudi Arabia; d Anatomic Pathology Department, King Faisal Specialist Hospital and Research Center, Riyadh, Saudi Arabia

**Keywords:** Orbit, Leiomyoma, Müller's muscle, Smooth muscle, Histopathology

## Abstract

**Introduction:**

Orbital leiomyoma can occur anywhere within the orbit. Posterior tumors arise from vessel wall smooth muscle cells, while anterior tumors develop from the capsulopalpebral or Muller muscle.

**Presentation of case:**

we present a case of a 24-year-old man who was referred for evaluation of chronic right eye swelling involving the medial upper eyelid. Ophthalmologic examination was unremarkable apart from upper lid fullness along with downward and lateral displacement of the globe. MRI revealed a large and well-defined growth in the right medial orbital space. An excisional biopsy was performed which showed a friable mass strongly adherent to the underlying conjunctiva and Muller muscle. This case report has been prepared and reported in line with the SCARE criteria.

**Discussion:**

Orbital leiomyoma usually presents with progressive painless proptosis if situated deep in the orbit. Our patient presented mainly with eyelid fullness and displacement due to the anterior location of his tumor. The diagnosis of orbital leiomyoma has to be confirmed by histopathological and immunohistochemical assessments such as in our case. A literature review was conducted using multiple databases spanning from 1963 to 2023 which concluded male predominance. One similar case to ours has been reported with the tumor originating from supraorbital neurovascular bundle.

**Conclusion:**

To the best of our knowledge, this is the first reported case of an orbital leiomyoma originating from the Muller muscle in the English literature.

## Highlights

• Orbital leiomyoma is a benign tumor of variable muscle origin depending on the location.
• Histopathology and immunohistochemistry are essential for the diagnosis.
• This is a unique case of anterior Orbital leiomyoma arising from Muller muscle.
• Surgical excision is the golden method of treatment.

## 1 Introduction

Leiomyoma, a benign spindle-cell tumor that commonly affects the uterus and gastrointestinal system, is rare in the orbit [1,2]. The tumor is presumably derived from vascular smooth muscle cells and possible sources in the orbit include orbital blood vessels, Muller's muscle or capsulopalpebral muscle of Hessar [3]. A common clinical presentation is a painless and slowly progressive proptosis [4].

A literature review was performed on several databases including PubMed, Google Scholar and Cochrane Library. The paper selection period spanned literature from 1963 to 2023 and search results were limited to articles published in the English-written language. The keyword search included Orbital, Leiomyoma, Muller Muscle, Muller's Muscle, Clinical manifestation and Immunohistochemistry. The selected articles were reviewed to assess their relevance, numbers of cases reported, and quality.

We report a patient that presented with upper eyelid fullness that prompted a clinical investigation that led to the diagnosis of primary orbital leiomyoma. Our case is interesting because of the anatomic location of the tumor which has not been reported before in the English literature. This case report has been prepared and reported in line with the SCARE criteria [5].

## 2 Presentation of case

A 24-year-old man presented to our oculoplastic service with gradual onset of right eye swelling involving the medial part of the upper eyelid for over 10 years. He had no associated pain, redness, diplopia, or blurred vision. There was no history of head or facial trauma and past medical history was clear of any chronic conditions.

**Fig. 1:**
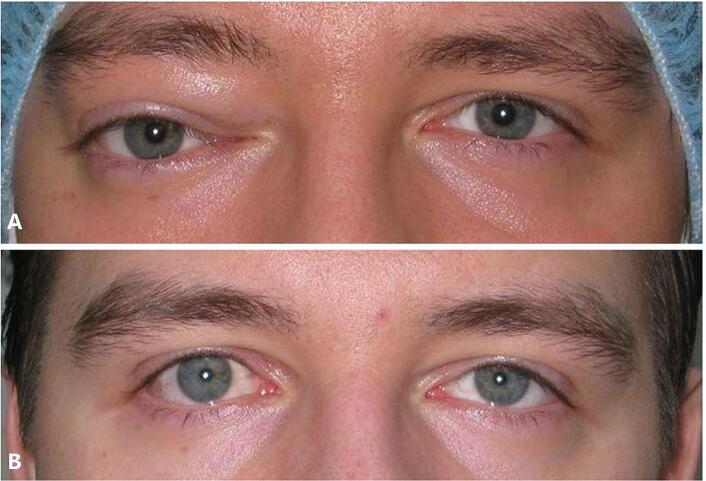
Pre-operative photo (in **A)** and post-operative photo (in **B**) showing right upper eyelid fullness with a downward and lateral displacement of the right globe. Note the resolution of globe displacement and upper eyelid swelling in B.

On examination, his vital signs were normal and best corrected visual acuity was 20/20 in each eye. Extraocular movements were full in all gazes with no restrictions. Upper eyelid fullness with downward and lateral displacement of the right globe was noted (Fig. 1A). Both pupils were equal and reactive without a relative afferent pupillary defect. Intraocular pressures were normal in both eyes. Anterior segment examination was unremarkable and posterior segment examination showed normal fundi and optic discs in both eyes.

**Fig. 2:**
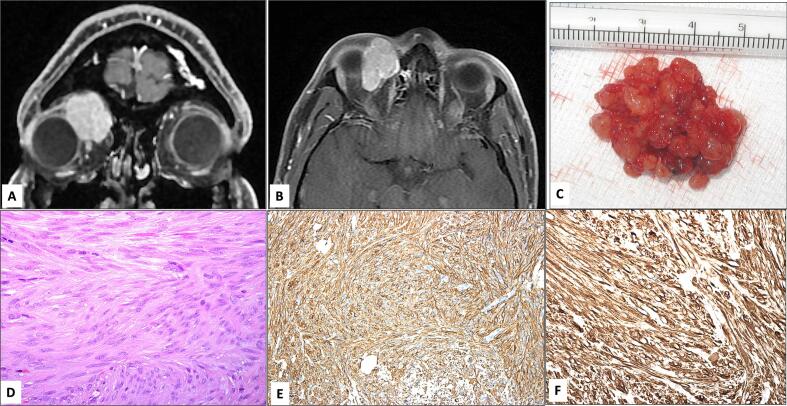
**A & B**: Magnetic Resonance Imaging showing T1-weighted coronal (in A) and axial (in B) cuts with contrast and fat suppression showing diffuse contrast enhancement of the medial orbital lesion along with downward and lateral globe displacement. **C**: The gross appearance of the excisional biopsy of the medial orbital lesion showing multiple lobulated friable tissue masses. **D**: The histopathological appearance of the tumor showing wavy bundles of spindle cells (Original magnification ×400 Hematoxylin and eosin). **E & F**: The immunohistochemical staining of the tumor cells expressing reactivity to Smooth muscle actin (SMA) in E and Desmin in F (Original magnification ×200 SMA and ×400 Desmin).

MRI revealed a large, partially lobulated, and well-defined lesion in the right medial orbital space measuring 1.95 × 3.16 × 2.62 cm (Fig. 2A & B). The lesion was isointense relative to recti muscles on T1-weighted images and hyperintense with homogeneous enhancement on contrast media. The lesion occupies the anterior superior medial extraconal space and extends posteriorly to the intraconal space resulting in inferior and temporal displacement of the globe.

The patient underwent an anterior orbitotomy via a lid crease incision. Upon accessing the superior medial orbital space, the mass was found to be adherent to the underlying conjunctiva and attached to the superior border of the upper the tarsus medially. Multiple lobulated friable tissue masses were visualized (Fig. 2C) and it was noted to be attached to the central part of the Muller muscle. Then the mass was removed completely. The mass bled profusely throughout its excision. The patient received oral prednisolone and topical tobramycin-dexamethasone 0.3 %–0.1 % for 1 week.

Histopathological studies revealed monomorphic spindle cell neoplasm with cigar-shaped nuclei and dispersed foci of calcification but no significant mitotic activity or necrosis (Fig. 2D). Tumor cells showed positive staining for smooth muscle actin (SMA) and desmin (Fig. 2E & F); negative for S100 protein and CD34, and a diagnosis of leiomyoma with secondary calcification was confirmed. At the postoperative month 4 appointment, his visual acuity remained unchanged, and his globe displacement resolved with no evidence of recurrence (Fig. 1B), however, the patient didn't show up for a follow up radiology imaging as he resides outside the country.

## 3 Discussion

Orbital leiomyoma represents a gradually developing benign neoplasm that arises from smooth muscle and can manifest at various sites within the orbital region [6]. It is proposed that anterior tumors may have their origin in the capsulopalpebral or Muller muscle, while posterior tumors could arise from the smooth muscle cells present in vessel walls [7]. Clinically, the most prevalent presentation is characterized by the insidious progression of painless proptosis but the diagnosis remains challenging owing to the rarity of such a neoplasm in this location [6,8]. The treatment modality of choice involves complete surgical excision, with radiation therapy being contraindicated due to its resistance to radiotherapy and the potential for radiation-induced sarcoma [6,9]. However, successful treatment of a non-circumscribed orbital leiomyoma with the gonadotropin-releasing hormone (GnRH) analog has been reported in the literature [10].

Histopathological analysis alone may be insufficient for establishing a definitive diagnosis of orbital leiomyoma. This inadequacy stems from the fact that the histopathological features bear resemblance to those of other spindle cell tumors within the orbit, including schwannoma, neurofibroma, fibrous histiocytoma, and solitary fibrous tumor [11]. The incorporation of immunohistochemical studies plays a pivotal role in reaching a definitive diagnosis. Histopathological studies reveal bundles of elongated spindle cells with eosinophilic cytoplasm and blunt-ended, cigar-shaped nuclei, while immunohistochemical investigations indicate immunoreactivity to smooth muscle actin, desmin, and vimentin [8,11]. Emphasis should be placed on the absence of nuclear pleomorphism, hyperchromatism, mitotic figures, or nuclear atypia in order to effectively differentiate leiomyoma from its malignant counterpart, leiomyosarcoma [7].

A review of previously reported cases of orbital leiomyoma has revealed a male predilection. In most cases, including our own, patients with anteriorly located orbital tumors presented with a visible eyelid mass. In contrast, patients with posteriorly located orbital tumors typically presented with proptosis [11].

In our case, the patient presented with an upper eyelid mass occupying the anterior superior medial extraconal space and extending posteriorly. MRI indicated that the mass was hypo- to isointense relative to the recti muscles on T1-weighted images. During surgical excision, the mass was found to be strongly adherent to the underlying conjunctiva, tarsus, and Muller muscle. Sendul et al. reported a similar case but with an orbital leiomyoma originating from the supraorbital neurovascular bundle [12]. To the best of our knowledge, there are no reported cases of orbital leiomyoma originating from the Muller muscle, making our case particularly intriguing.

## 4 Conclusion

When dealing with well-defined orbital masses, it is advisable to consider leiomyomas in the differential diagnosis. Complete surgical excision remains the treatment of choice. Diagnosing leiomyoma based solely on histopathological studies can be challenging, given the similarities with other benign spindle cell tumors under light microscopy. Thus, immunohistochemical studies provide the most reliable method for establishing a definitive diagnosis.

## Provenance and peer review

Not commissioned, externally peer reviewed.

## Consent

Written informed consent was obtained from the patient for publication and any accompanying images. A copy of the written consent is available for review by the Editor-in-Chief of this journal on request.

## Ethical approval

An ethical approval is not required for case reports as per the regulations of the Institutional Review Board, College of Medicine, King Saud University. However, information was obtained and reported in a manner that was compliant with the standards set forth by the Health Insurance Portability and Accountability Act, and the Declaration of Helsinki as amended in 2013.

## Guarantor

Hind M. Alkatan

## Research registration number

Not applicable.

## Funding

This research did not receive any specific grant from funding agencies in the public, commercial, or not-for-profit sectors.

## Author contribution

**Rakan Alsaad**: acquisition of data and drafting of the manuscript; **Hind M. Alktan**: critical review of the manuscript for submission; **Hatim Khoja**: Histopathological diagnosis; **Adel Alsuhaibani**: concept and design of the study as well as the final approval of the version.

## Conflict of interest statement

None.
